# On Credibility, Clarity, and Compliance

**DOI:** 10.1074/mcp.E115.052506

**Published:** 2015-06-03

**Authors:** Al Burlingame, Steven A. Carr, Ralph A. Bradshaw, Robert J. Chalkley

Since its inception, MCP has recognized the promise of proteomics and its underlying technologies to significantly move the boundaries of knowledge in biology and medicine. As has been noted (1), proteomics represented a paradigm shift in how experiments were designed and executed and how the resultant data were interpreted and stored. But the journal has also recognized that the value of the proteomic approach and the data generated were only as good as the quality and reliability of that data—a corollary of the computational maxim, “garbage in, garbage out.” Thus, in its very earliest stages, the editorial staff of MCP, particularly Steve Carr, Ruedi Aebersold, and Al Burlingame, began earnest discussions about how a journal should evaluate data arising from large scale, often called “shotgun,” mass spectrometric experiments, which ultimately lead to the first set of guidelines (2). Initially, these questions focused on correctly identifying peptide sequences (as surrogates for the proteins they were derived from) but quickly expanded to the identification and localization of post-translational modifications (PTMs) and mass-spectrometric-based quantification as well (3, 4). In the main, it was decided that manuscripts submitted to the journal should provide sufficient information to allow an appropriate assessment by first reviewers and then readers. At first, the requested information consisted of the methods used for collection and interpretation (basically search parameters) of the data as well as the identifications themselves. As the guidelines evolved, tutorials and checklists were introduced to aid the submission process. The latter were eventually expanded to include checklists for papers of clinical relevance and those reporting glycomic analyses (5, 6). Finally, after first introducing the guidelines simply as recommendations, the journal began enforcing them by subjecting all submitted articles to a review to determine if they were compliant. The compliance check is not, and was never meant to be, a part of the evaluative peer review process, as these checks only determine whether the necessary information has been supplied and do not assess in any way its quality or interpretation.

One of the most challenging aspects of the documentation process as it developed was the requirement that authors provide “annotated” spectra for all MS/MS protein identifications based on only a single unique peptide (including the spectra used in peptide mapping fingerprints (PMFs)) and all PTM assignments. Annotated spectra means the labeling of the *m/z* for all significant peaks in the spectra as well as their fragment ion designations (*e.g.* y, b, etc. if spectra are from an MS/MS experiment) relative to the sequence being reported. Given the variations in software supporting different instruments and in the outputs of the many individual search engines, this single requirement became an increasingly difficult one to manage. In keeping with the historical “maintenance of the public record” role, which basically requires journals to report all the data that are germane to the claims and conclusions of an article, MCP at the outset considered that these spectra needed to be submitted with the article and be a part of the journal. Electronic publishing of biological journals, which began with the *Journal of Biological Chemistry* in 1995 (7), made this possible, at least at first. However, when very large-scale identifications of PTMs became commonplace, the problem was substantially exacerbated, and it became clear that, at least in some cases, it was impractical to insist that these spectra be submitted as supplemental material. In addition, many authors were meeting this compliance requirement through the production of one large pdf file of static screenshots of spectra, a format that made finding relevant spectra and more than cursory examination of the assignment difficult and reanalysis of data impossible.

At the same time, the journal was also wrestling with the issue of raw data and whether it should require authors to also make this information available. It is germane to note that these were two separate issues: The spectra were considered to be a part of the manuscript, while the raw data were not. However, they were certainly interrelated. Thus, as a compromise, and in recognition of the importance of supporting repositories that store data in a secure form while allowing full public access, MCP waived the requirement that spectra be submitted with the article, allowing deposition in an acceptable public repository in lieu of inclusion in the manuscript as an alternative. At the same time, it announced that it would require all raw data to be deposited in a similar public site. Unfortunately this led, in many cases, to the often incorrect conclusion that simply depositing the raw data met both requirements without appreciating the different goals of the two submissions: one providing the ability to easily assess the authors' interpretation of their data, the other allowing reanalysis and reuse of acquired data. This well-meaning decision to require the deposition of raw data subsequently hit “a serious snag” when one of the major sites available at the time began to experience very substantial problems, and when it became clear that some data that had been deposited there were becoming corrupted, the editors of MCP declared a moratorium on this requirement.

For the last year, the journal has spent a considerable amount of time examining its guidelines and the problems that authors have encountered in attempting to comply with them. Specifically, we focused on the requirements for annotated mass spectra, including the availability of appropriate viewers for search engine output formats, the deposition of raw data, and the efficiency of compliance checking. As these discussions developed, it was clear that there were several important facts to consider: 1) a review of the compliance records revealed, rather disappointingly, that a substantial number of submitted manuscripts still fail one or more of the checks, with the vast majority of these involving the MS requirements, and that this situation has not improved significantly since the journal started to enforce compliance; 2) there had been a clear uptick in the number of repositories that would accept both raw data and/or spectra; 3) the availability of suitable viewers to support a significant number of output files had increased significantly, and 4) the guidelines and supporting documents provided by the journal on the submission website were severely out of date and/or uninformative. In dealing with these issues, it was important to remember that the principal purpose of these guidelines is to ensure, to the extent possible, the correctness of the results and to provide for the clear and lucid interpretation of that data. It has been understood by the journal from the outset that data that appear in it (and any other journal for that matter) will inevitably find its way into other reference material, particularly web-based databases, and the reliability of this information is only as good as the sources from which it is derived. This alone is sufficient reason to upgrade the journal's guidelines that are designed, after all, for that purpose but making it easier for authors to submit to MCP is equally important (to us).

To address these problems, the journal has clarified its instructions to authors, introduced several changes in the submission site, upgraded its policy regarding raw data, and appointed a data management editor whose responsibilities will be to assess any continued issues related to compliance checking, monitor advances in data management (and suggest changes to the journal editorial staff to deal with them as they arise), and be available to assist authors in meeting the journal's requirements. Robert Chalkley of UCSF has accepted this assignment. He will work with the compliance checkers, associate editors, and authors to resolve any conflicts encountered in meeting the journal's requirements.

A number of common problems that were uncovered in the compliance review include:

- Failure to provide date (or version) of software used in analyzing data.
- Failure to provide date (or version) of the database searched (and the number of entries in it).
- Failure to provide the number of peptides and percentage coverage for each protein identified (this information must be include in the manuscript or supplemental data; placing it only in a repository in not acceptable).
- Failure to provide any user ID/password for deposited data (to enable reviewer access).
- Utilizing a laboratory or institutional website to deposit data; placing raw or processed data on such a site *in addition* to a public site is allowable but not as the sole site.

In order to help authors avoid these pitfalls, amended guidelines and instructions to authors have been added to the journal website to alert authors to these potential problem areas before submission. Among the most important additions is a description of what authors need to provide to allow the viewing of annotated spectra, depending on what software was used to interpret and process the original data. Consulting this tutorial should save considerable time and prevent unnecessary delays if a manuscript contains PTM identifications or proteins identified on the basis of a single peptide. It should perhaps be emphasized that the spectra requirements for PTMs do not extend to peptides containing oxidized methionine or other modifications introduced for quantification or stabilization.

Particularly with the increasing submission of quantitative studies, one area of the guidelines that it was felt needed to be strengthened is the description of the experimental design. Hence, in the revised guidelines a requirement for a separate paragraph that clearly delineates numbers of replicates, whether they are biological or technical repeats, and justification for why this experimental design has enough statistical power to infer biological conclusions has been added.

Finally, the journal has decided that it is time to restore the requirement for raw data deposition. There are now several repositories available that provide various levels of data storage, particularly those connected with the proteomeXchange consortium (8). Consulting with them directly or with the data management editor is encouraged if one is not familiar with these sites. This requirement is in keeping with what the journal perceives is the general sentiment of the community at large and is clearly supported by various stakeholders in the collection, analysis, funding, and storage of proteomic data (9). This policy will go into effect **July 1, 2015** and will require that the raw data be deposited at the time of submission of the manuscript. Providing the appropriate information describing this deposition in the cover letter or in the manuscript itself will be a prerequisite for further review.
